# Supplementary material for: Omissions of threat trigger subjective relief and prediction error-like signaling in the human reward and salience systems
Source: eLife. 2025 Feb 26;12:RP91400. doi: 10.7554/eLife.91400 (PMC11875134; doi:10.7554/eLife.91400)
Supplement: Supplementary file 1. [file elife-91400-supp1.docx]

**Supplementary File 1**

*Demographics of included participants*

| **Subject ID** | **Age** | **Gender** | **Excluded from rating analysis** | **Excluded from SCR analysis** | **Excluded from fMRI analysis** |
| --- | --- | --- | --- | --- | --- |
| Sub-01 | 24 | Male |  |  |  |
| Sub-02 | 19 | Female |  |  |  |
| Sub-03 | 21 | Female |  |  | Run 1 |
| Sub-04 | 25 | Female |  |  |  |
| Sub-05 | 19 | Female |  |  |  |
| Sub-06 | 20 | Male |  |  |  |
| Sub-07 | 19 | Female |  |  |  |
| Sub-08 | 24 | Female |  |  |  |
| Sub-09 | 22 | Male |  |  |  |
| Sub-10 | 21 | Female |  | All runs (non-responder) |  |
| Sub-13 | 20 | Male |  | All runs (non-responder) |  |
| Sub-14 | 20 | Male | Run 4 | Run 4 | Run 4 |
| Sub-15 | 19 | Female |  | First 4 trials of Run 1 |  |
| Sub-16 | 18 | Male |  |  |  |
| Sub-20 | 25 | Female |  |  |  |
| Sub-21 | 19 | Male | Run 4 | All runs (technical difficulties) | Run 4 |
| Sub-22 | 20 | Female |  | All runs (non-responder) |  |
| Sub-23 | 23 | Female |  |  |  |
| Sub-24 | 25 | Male |  |  |  |
| Sub-25 | 25 | Male |  | All runs (non-responder) |  |
| Sub-26 | 25 | Male |  |  |  |
| Sub-27 | 22 | Female |  |  |  |
| Sub-29 | 19 | Female |  |  |  |
| Sub-30 | 19 | Female |  |  |  |
| Sub-31 | 18 | Female |  |  |  |
| Sub-32 | 18 | Male |  |  |  |
| Sub-33 | 19 | Female | Last trial of Run 4 | Last trial of Run 4 | Run 4 |
| Sub-34 | 18 | Female |  |  |  |
| Sub-35 | 18 | Male |  |  |  |
| Sub-36 | 18 | Female |  |  |  |
| Sub-37 | 18 | Female |  |  |  |
| **TOTAL**  **(N = 31)** | ***M = 20.65*** | **19 Females** | **Final sample:**  **N = 31** | **Final sample:**  **N = 26 (17 females)** | **Final sample:**  **N = 31** |

*Note:* 6 additional participants were screened but were excluded because of technical issues during the scanning session resulting in absent and/or incomplete datasets (N = 3), or because they dropped out before the scanning session took place (N = 3).
