# Supplementary material for: Omissions of threat trigger subjective relief and prediction error-like signaling in the human reward and salience systems
Source: eLife. 2025 Feb 26;12:RP91400. doi: 10.7554/eLife.91400 (PMC11875134; doi:10.7554/eLife.91400)
Supplement: Supplementary file 2. [file elife-91400-supp2.docx]

**Supplementary File 2**

*Descriptives of questionnaire scores*

| **Questionnaire scale** | **Mean** | **Standard deviation** | ***N*** |
| --- | --- | --- | --- |
| STAI Trait | 36.68 | 9.25 | 31 |
| IUS | 63.20 | 13.46 | 30 |
| Prospective subscale | 17.47 | 4.95 | 30 |
| Inhibitory subscale | 10.20 | 3.36 | 30 |
| DTS | 3.69 | 0.67 | 31 |
| Absorption subscale | 3.59 | 0.87 | 31 |
| Appraisal subscale | 3.85 | 0.77 | 31 |
| Regulation subscale | 3.46 | 0.79 | 31 |
| Tolerance subscale | 3.87 | 0.68 | 31 |
| DASS |  |  |  |
| Depression subscale | 7.00 | 6.23 | 30 |
| Anxiety subscale | 2.97 | 3.58 | 30 |
| Stress subscale | 7.43 | 5.38 | 30 |
| ASI | 14.97 | 8.16 | 30 |
| Physical subscale | 4.10 | 3.40 | 30 |
| Cognitive subscale | 3.23 | 3.45 | 30 |
| Social subscale | 7.63 | 4.07 | 30 |
| LOTR |  |  |  |
| Optimism subscale | 7.10 | 2.49 | 31 |
| Pessimism subscale | 5.19 | 2.51 | 31 |
| BAS |  |  |  |
| Drive subscale | 11.45 | 2.03 | 31 |
| Fun seeking subscale | 12.03 | 2.01 | 31 |
| Reward responsiveness subscale | 17.26 | 1.95 | 31 |
| BIS | 20.71 | 3.73 | 31 |
| PANAS |  |  |  |
| Positive subscale | 37.10 | 3.84 | 31 |
| Negative subscale | 21.71 | 5.72 | 31 |
| PCS |  |  |  |
| Helplessness subscale | 4.50 | 2.96 | 30 |
| Magnification subscale | 2.90 | 1.97 | 30 |
| Rumination subscale | 7.73 | 2.78 | 30 |

*Note.* Participants filled out a questionnaire battery during the intake session, as part of a larger attempt to relate individual differences in anxiety- and pain-related traits to individual differences in relief. The battery consisted of Dutch versions of the Depression, Anxiety & Stress Scales (DASS)(De Beurs & Van Dyck, 2001; Lovibond & Lovibond, 1995), State-Trait Anxiety Inventory (STAI)(Spielberger et al., 1983; Van der Ploeg, 1982), Intolerance of Uncertainty Scale (IUS)(de Bruin et al., 2006; Freeston et al., 1994), Positive and negative Affect Schedule (PANAS)(Engelen et al., 2006; Watson et al., 1988), Behavioral Inhibition Scale and Behavioral Activation Scale (BIS/BAS)(Franken et al., 2005; White & Carver, 1994), Distress Tolerance Scale (DTS)(Simons & Gaher, 2005) and Life Optimism Trait – Revised (LOT-R)(Klooster et al., 2010; Scheier et al., 1994), the Anxiety Sensitivity Index (ASI)(Taylor et al., 2007), and the Pain Catastrophizing Scale (PCS)(Sullivan et al., 1995). *N* represents number of participants that completed the questionnaire and that was used to calculate the summary statistics.

References

De Beurs, E., & Van Dyck, R. (2001). De DASS: een vragenlijst voor het meten van depressie, angst en stress. *Gedragstherapie : Tijdschrift Voor Gedragstherapie En Cognitieve Therapie.*, *34*, 35–53.

de Bruin, G. O., Rassin, E., van der Heiden, C., & Muris, P. (2006). Psychometric properties of a Dutch version of the Intolerance of Uncertainty Scale. *Netherlands Journal of Psychology*, *62*(2), 87–92. https://doi.org/10.1007/BF03061055

Engelen, U., De Peuter, S., Victoir, A., Van Diest, I., & Van den Bergh, O. (2006). Verdere validering van de positive and negative affect schedule (PANAS) en vergelijking van twee Nederlandstalige versies. *Gedrag En Gezondheid*, *34*(2), 89–102.

Franken, I. H. A., Muris, P., & Rassin, E. (2005). Psychometric properties of the Dutch BIS/BAS scales. *Journal of Psychopathology and Behavioral Assessment*, *27*(1), 25–30. https://doi.org/10.1007/s10862-005-3262-2

Freeston, M. H., Rhéaume, J., Letarte, H., Dugas, M. J., & Ladouceur, R. (1994). Why do people worry? *Personality and Individual Differences*, *17*(6), 791–802. https://doi.org/10.1016/0191-8869(94)90048-5

Klooster, P. M. ten., Weekers, A. M., Eggelmeijer, F., van Woerkom, J. M., Drossaert, C. H. C. M., Taal, E., Rasker, J. J., & Baneke, J. J. (2010). Optimisme en/of pessimisme: factorstructuur van de Nederlandse Life Orientation Test-Revised. *Psychologie En Gezondheid*, *38*(2), 89–100. https://doi.org/10.1007/BF03089356

Lovibond, P. F., & Lovibond, S. H. (1995). The structure of negative emotional states: Comparison of the Depression Anxiety Stress Scales (DASS) with the Beck Depression and Anxiety Inventories. *Behaviour Research and Therapy*, *33*(3), 335–343. https://doi.org/https://doi.org/10.1016/0005-7967(94)00075-U

Scheier, M. F., Carver, C. S., & Bridges, M. W. (1994). Distinguishing Optimism From Neuroticism (and Trait Anxiety, Self-Mastery, and Self-Esteem): A Reevaluation of the Life Orientation Test. *Journal of Personality and Social Psychology*, *67*(6), 1063–1078. https://doi.org/10.1037/0022-3514.67.6.1063

Simons, J. S., & Gaher, R. M. (2005). The distress tolerance scale: Development and validation of a self-report measure. *Motivation and Emotion*, *29*(2), 83–102. https://doi.org/10.1007/s11031-005-7955-3

Spielberger, C., Gorsuch, R., Lushene, R., Vagg, P. R., & Jacobs, G. (1983). Manual for the State-Trait Anxiety Inventory (Form Y1 – Y2). In *Palo Alto, CA: Consulting Psychologists Press; Vol. IV*.

Sullivan, M. J. L., Bishop, S. R., & Pivik, J. (1995). The Pain Catastrophizing Scale: Development and validation. *Psychological Assessment*, *7*(4), 524–532. https://doi.org/https://doi.org/10.1037/1040-3590.7.4.524

Taylor, S., Zvolensky, M. J., Cox, B. J., Deacon, B., Heimberg, R. G., Ledley, D. R., Abramowitz, J. S., Holaway, R. M., Sandin, B., Stewart, S. H., Coles, M., Eng, W., Daly, E. S., Arrindell, W. A., Bouvard, M., & Cardenas, S. J. (2007). Robust Dimensions of Anxiety Sensitivity: Development and Initial Validation of the Anxiety Sensitivity Index-3. *Psychological Assessment*, *19*(2), 176–188. https://doi.org/10.1037/1040-3590.19.2.176

Van der Ploeg, H. M. (1982). De zelf-beoordelings vragenlijst (STAY-DY). *Tijdschrift Voor Psychiatrie*, *24*, 576–588.

Watson, D., Clark, L. A., & Tellegen, A. (1988). Development and validation of brief measures of positive and negative affect: The PANAS scales. *Journal of Personality and Social Psychology*, *54*(6), 1063–1070.

White, T. L., & Carver, C. S. (1994). Behavioral inhibition, behavioral activation, and affective responses to impending reward and punishment: The BIS/BAS scales. *Journal of Personality and Social Psychology*, *67*(2), 319–333. http://www.psy.miami.edu/faculty/ccarver/sclBISBAS.html%5Cnhttp://www.scribd.com/doc/8760706/Carver1994?secret_password=zkhvhvj8dknjmpqjutf
