# Supplementary material for: Omissions of threat trigger subjective relief and prediction error-like signaling in the human reward and salience systems
Source: eLife. 2025 Feb 26;12:RP91400. doi: 10.7554/eLife.91400 (PMC11875134; doi:10.7554/eLife.91400)
Supplement: Supplementary file 3. [file elife-91400-supp3.docx]

**Supplementary File 3**

*Trial types and numbers*

***
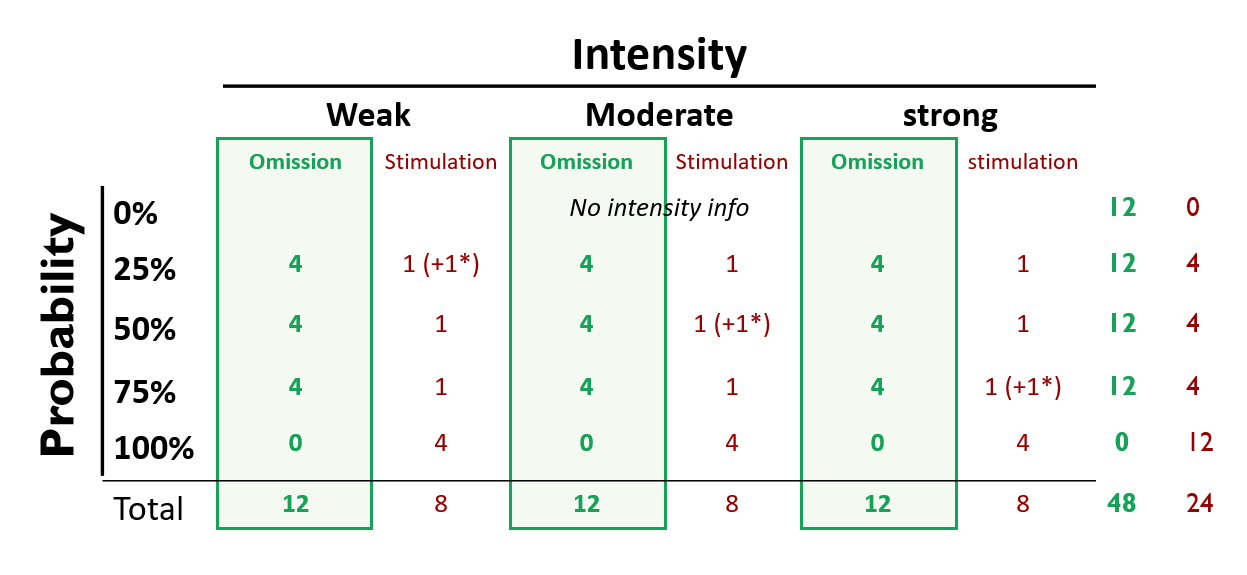
***

** This is an example. In general there should be one additional weak, one additional moderate, and one additional strong stimulation with exactly one having probability 25%, one having probability 50%, and one having probability 75%*
